# Unusual Aspergillus Pleural Effusion in a Patient With Immunodeficiency

**DOI:** 10.7759/cureus.46308

**Published:** 2023-10-01

**Authors:** Ked Fortuzi, Sneha Khanal, Patrik Schmidt, Tanushree Bhatt, Misbahuddin Khaja

**Affiliations:** 1 Pulmonary Medicine, Bronx Care Health System, New York, USA; 2 Internal Medicine, Bronx Care Health System, New York, USA

**Keywords:** liver transplant, isavuconazole, immunocompromised, pleural effusion, pulmonary aspergillosis

## Abstract

Pleural aspergillosis is a rare form of invasive bronchopulmonary aspergillosis that is most often seen in immunocompromised hosts. It appears because of the coagulative necrosis of lung tissue induced by the *Aspergillus *species, which promotes the formation of a fungal pleural effusion. We present the case of a 51-year-old liver transplant patient on chronic immunosuppression therapy who presented with respiratory failure and was found to have a large left-sided pleural effusion from invasive aspergillosis. After thoracentesis, he started antifungal therapy with isavuconazole. This newer, second-generation broad-spectrum triazole is non-inferior to voriconazole but with less hepatotoxicity and was noted to have an improvement in his symptoms. In the differential diagnosis of pulmonary effusions in immunocompromised patients, it is crucial to consider invasive aspergillosis, as demonstrated by our case. This case study highlights the importance of quick diagnosis and treatment to enhance outcomes in this vulnerable population.

## Introduction

Pulmonary aspergillosis has various presentations, including allergic bronchopulmonary aspergillosis, aspergilloma, necrotizing aspergillus pneumonia, and invasive aspergillosis ​[1]. Pleural aspergillosis is a form of invasive aspergillosis that occurs due to infection with *Aspergillus fumigatus* or *Aspergillus flavus*, with or without lung involvement ​[2].​ It usually occurs in the background of chronic lung pathologies, including infections or scarring of the lung, previous lung or pleural surgeries, or immunocompromised patients. Even among this subset of patients, pleural aspergillosis is an extremely rare presentation. Fungal pleural effusion accounts for less than 1% to 5% of pleural effusions ​[2,3]. *Aspergillus* pleural effusion is rare, and managing this disease process remains challenging ​[4].​ We present one such rare presentation of this potentially fatal disease in a liver transplant recipient under immunosuppressive therapy.

## Case presentation

Emergency medical services brought a 51-year-old male to the emergency department with complaints of generalized abdominal pain, shortness of breath at rest, and generalized back pain for one week. He also reported having nausea, vomiting, diarrhea, dysuria, and hematuria for five days prior to the presentation. His medical history included long-standing chronic hypertension, type 2 diabetes mellitus, and recent acute liver failure secondary to hepatitis B exacerbated by acetaminophen toxicity, for which he underwent an emergency liver transplant six months prior to presentation. His postoperative course was complicated by bleeding, acute kidney injury, and an Escherichia coli and Enterobacter wound infection. Following the transplant, the patient endorsed taking immunosuppressive medications, including mycophenolate mofetil and tacrolimus. His other home medications included entecavir, aspirin, atorvastatin, pantoprazole, gabapentin, and sitagliptin. However, his exact medication dosage and frequency were not able to be confirmed during his admission. He denied any allergies, smoking, alcohol use, or use of any toxic substances.

**Figure 1:**
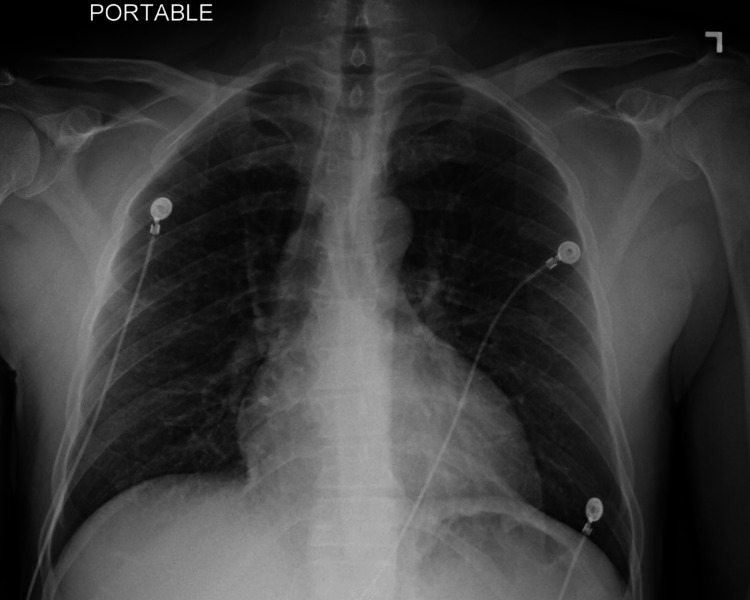
Patient’s chest X-ray one year prior to presentation The patient’s previous chest X-ray showed no anomalies of acute significance. Please note the absence of pleural effusions in this chest X-ray.

**Figure 2:**
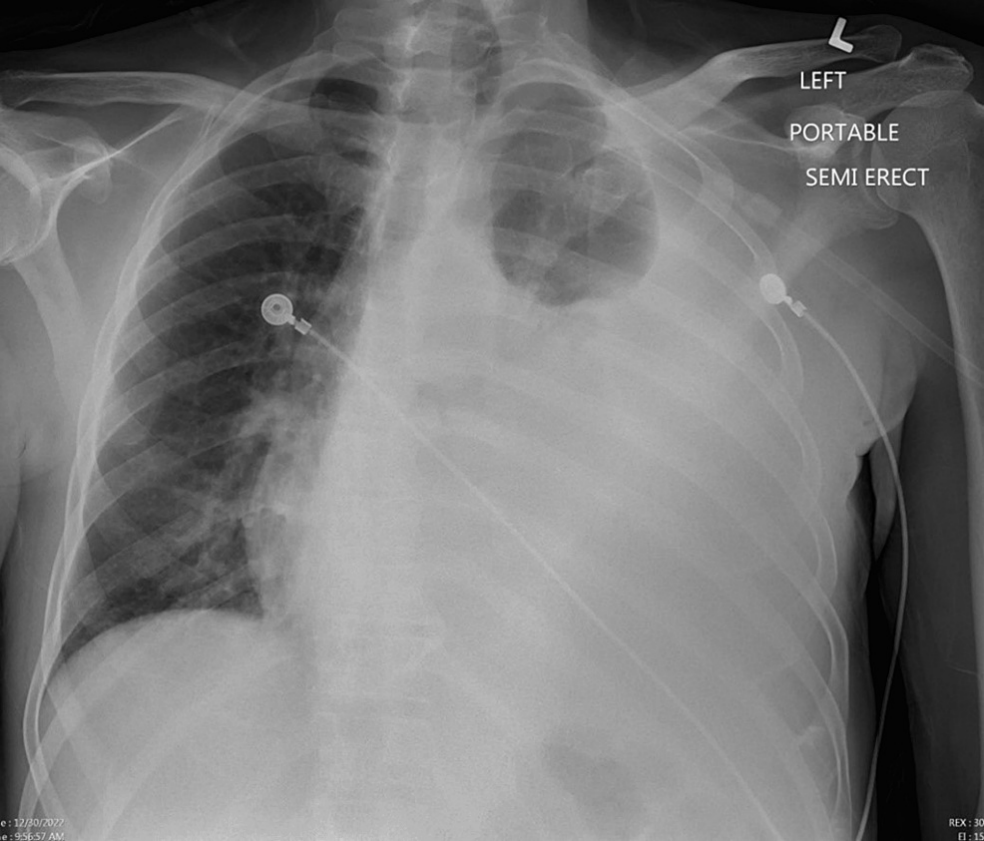
A chest X-ray demonstrating large volume left-sided pulmonary effusion with mass effect resulting in a midline shift of mediastinal structures and trachea.

**Figure 3:**
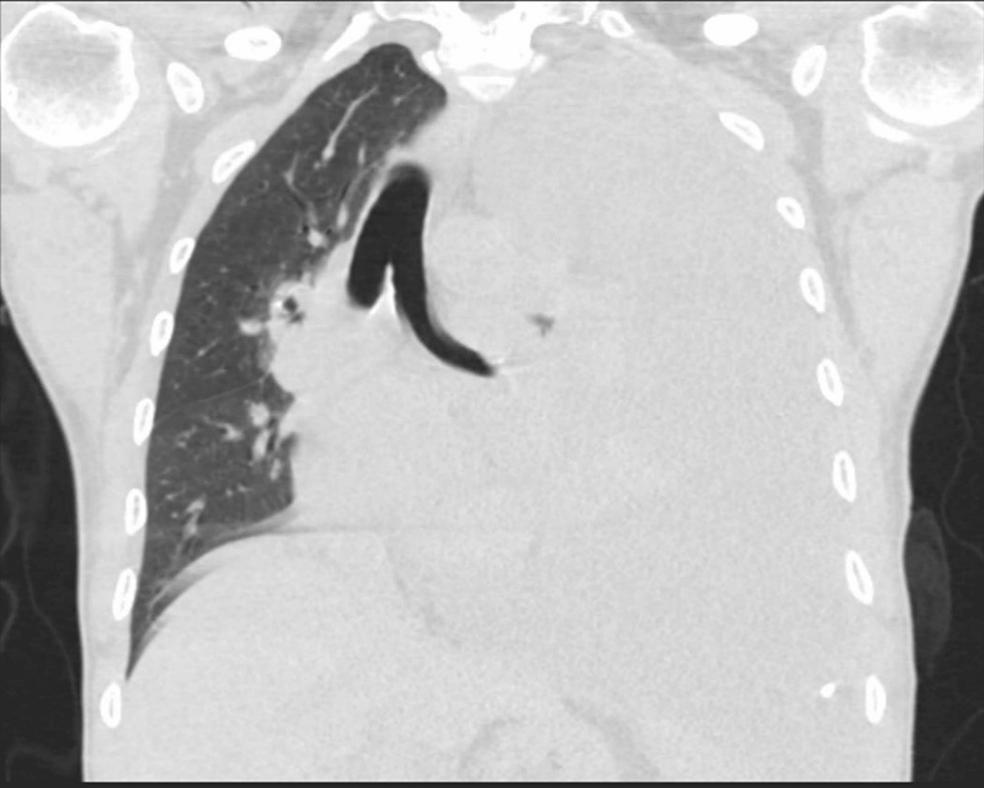
CT chest demonstrating large left-sided pleural effusion with mass effect pushing the mediastinal structures to the right side (coronal view)

**Figure 4:**
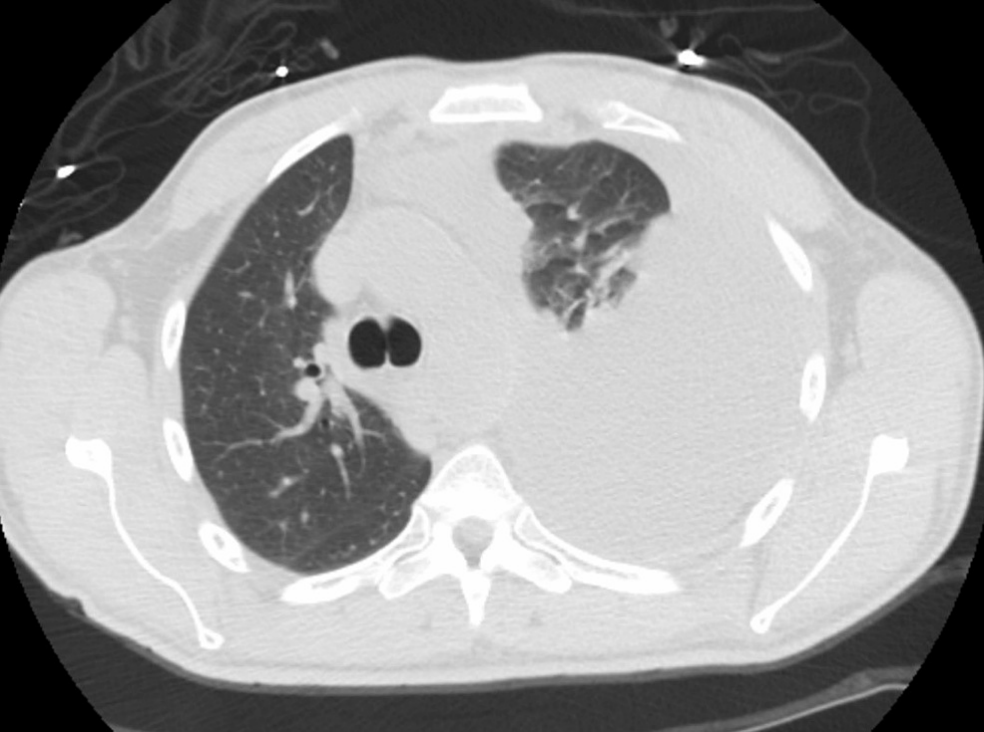
CT chest demonstrating large left-sided pleural effusion with mass effect pushing the mediastinal structures to the right side (axial view)

In the emergency department, vital signs were as follows: blood pressure 136/78 mm Hg, pulse 105 beats per minute, and respiratory rate at 28 breaths per minute. The patient was in respiratory distress, using accessory muscles for respiration, and there were decreased breath sounds over the left anterior and posterior chest. He had abdominal distension with moderate tenderness to palpation over all quadrants, dullness of percussion in the flanks, and sluggish bowel sounds. Initial laboratory findings are detailed in Table 1, which were significant for anemia, hyperkalemia, and acute kidney injury. His liver function tests were found to be normal on presentation. A chest X-ray showed a large left-sided pleural effusion with a mediastinal shift to the right (Figures 1-2). A CT of the chest showed a large left-pleural effusion with a collapse of the underlying left lung and a mediastinal shift to the right (Figures 3-4). A CT of the abdomen showed a small bowel adynamic ileus and significant volume ascites. The patient underwent a diagnostic and therapeutic thoracentesis, during which 1.5L of fluid was drained, with analyses showing a lymphocytic exudative effusion (Tables 2-3). Blood cultures were sent; however, no growth was detected.

**Table 1:** Initial laboratory investigations upon presentation

Parameters	Values	Reference range
Hemoglobin	8.7 gm/dl	12-16 gm/dl
Hematocrit	27.4%	42-51%
White blood cells	5 K/Ul	4.8-10.8 K/Ul
Platelets	495 K/Ul	150-400 K/Ul
Blood urea nitrogen	39 mg/dl	8-26 mg/dl
Creatinine	2.3 mg/dl	0.5-1.5 mg/dl
Sodium	131 mEq/L	135-145 mEq/L
Potassium	5.5 mEq/L	3.5-5.0 mEq/L
Lactate dehydrogenase	353 U/L	100-190 U/L
Total protein	7.2 gm/dl	6-8.5 gm/dl

**Table 2:** Pleural fluid analysis results.

Parameters	Values	Reference Range
White blood cells	1520 cells/mm3	1.716 cells / mm3
Red blood cells	7250 million cells/mm3	0 – 1, 070 million cells / mm3
Segmented count	8%	40% -60%
Lymphocyte count	88%	20 %– 40 %
Albumin	2.4 gm/dl	3.4 - 5.4 gm / dL
Amylase	53 U/l	30 – 110 U/L
Cholesterol	67.8 mEq/L	200 – 239 mEq /l
Glucose	198 mg/dl	50 – 80 mg/dl
Lactate dehydrogenase	237 U/L	105 – 333 U/l
Protein	5 gm/dl	6.0 – 8.3 gm/dl

**Table 3:** Pleural fluid analysis results, cultures, ADA, and cytology ADA: Adenosine deaminase

Analysis of pleural fluid	Results
Gram Stain	Rare gram-positive bacilli
Mycobacteria	Negative
Adenosine Deaminase	23.6 U/L
Cytology	Negative (all three)
Fungal Cultures	Aspergillus fumigatus

After thoracentesis, the patient was promptly started on an antifungal agent, which in our case was isavuconazole. As he had significant improvement in his abdominal pain and shortness of breath after thoracentesis, our patient was discharged on a six-month course of isavuconazole, with close outpatient follow-up to augment the duration of therapy based on the disease course.

## Discussion

Aspergillosis is a group of fungal infections caused by the *Aspergillus* species, first described by John Cleland in 1924. Most infections are caused by *A. fumigatus*, *Aspergillus niger*, *A. flavus*, and *Aspergillus terreus* [5]. Pulmonary aspergillosis is a rare disorder that occurs when the pulmonary defense systems are disrupted in immunocompromised states such as hematologic malignancies, organ transplantations, acquired immunodeficiency syndrome (AIDS), or active immunosuppressant therapy [6]. Innate cellular immunity provided by the combined actions of phagocytes, airway epithelial cells, and alveolar macrophages serves as the basis for clearing fungal infections in the lung [7]. However, in individuals unable to clear the initial infection, invasion by *Aspergillus* leads to hemorrhage and coagulative necrosis of the lung tissue [8]. Rhinosinusitis and pulmonary aspergillosis are the most common types of invasive diseases, with chronic aspergillosis and allergic bronchopulmonary *Aspergillus *seen more commonly in patients with other concomitant respiratory conditions, such as asthma. Cutaneous forms of aspergillosis, while rarer, can also be seen through direct inoculation of a wound by *Aspergillus* species. [9] 

Pleural effusions without underlying lung disease are unusual occurrences. The initial presentation may vary; however, a classic triad of fever, pleuritic chest pain, and hemoptysis have been described in neutropenic patients with pulmonary aspergillosis [10]. Different CT scan findings are seen based on the type of aspergillosis. However, pulmonary nodules, patchy or segmental consolidation, and peri-bronchial infiltrates are often noted [11]. Confirmatory testing includes isolating the *Aspergillus* species from affected tissues or pleural fluid via thoracentesis by performing microscopic examinations, body fluid cultures, and sensitivity testing. 

Antifungal therapy is usually the primary treatment for patients with persistent and severe *Aspergillus* pleural effusions. Voriconazole is the first-line treatment for aspergillosis, followed by liposomal amphotericin B if the patient is not responding to the first-line agent [12]. Surgical intervention, such as necrotic tissue debridement, may also be considered in patients with chronic necrotizing disease [13]. Careful monitoring and follow-up are necessary to ensure optimal patient outcomes. In our patient with a liver transplant, voriconazole and amphotericin could have caused liver toxicity and were thus avoided. Isavuconazole is a newer antifungal agent with a lower hepatotoxic profile than the older and more traditional antifungal agents [14]. 

## Conclusions

Aspergillus pleural effusion is a rare clinical manifestation of invasive bronchopulmonary aspergillosis that can be a significant diagnostic challenge. Therefore, we need to develop a more standardized protocol for management. The lack of one makes it difficult to compare the outcomes of different agents used for its treatment. Therefore, further studies are needed to develop guidelines that will help diagnose and treat this condition.
